# Use of a Self-Irrigating Bipolar Sealer for Bilateral C2 Nerve Root Ligation During Atlantoaxial Instrumented Fusion

**DOI:** 10.7759/cureus.112293

**Published:** 2026-07-08

**Authors:** Steve S Cho, James J Zhou, Mark E Oppenlander, Daniel J Coughlin, Bryan S Lee

**Affiliations:** 1 Department of Neurosurgery, Barrow Neurological Institute, St. Joseph’s Hospital and Medical Center, Phoenix, USA; 2 HonorHealth Neuroscience Research and Innovation Institute, HonorHealth Medical Center, Scotsdale, USA; 3 Department of Neurosurgery, Madigan Army Medical Center, Tacoma, USA

**Keywords:** aquamantys, atlantoaxial fixation, bipolar sealer, c1 lateral mass, c2 nerve roots

## Abstract

Background

This research involves the analysis of a novel surgical technique using a commercial self-irrigating bipolar sealer for ligation of the C2 nerve-venous complex during atlantoaxial fixation.

Methods

For this retrospective analysis, surgical exposure of C1-2 was performed via an open approach. The C2 nerve root and perineural venous plexus were coagulated with the bipolar sealer at 100-140 watts for 10-30 seconds and cut using microscissors, fully exposing the C1-2 facet joints to allow instrumentation of the C1 lateral masses and C2 pars and pedicles under direct visualization. Arthrodesis was accomplished within the exposed C1-2 joint spaces.

Results

Data are presented as mean (standard deviation (SD)). Twenty-seven patients (15 men, 12 women) with a mean age of 71.6 (8.6) years and mean body mass index 28.6 (6.4) underwent atlantoaxial fixation and ligation of the C2 nerve roots, either in isolation or as part of longer constructs. The mean operative time was 126.4 (55.9) minutes; the estimated blood loss (EBL) was 82 (72) mL. For C1-2 fusion only, the mean operative time and EBL were 113.5 (55.9) minutes and 49 (25) mL, respectively. The mean time to complete exposure of the C1-2 joint was 2.3 (0.9) minutes. No perioperative or postoperative complications were noted, except C2 dermatome numbness without dysesthesia.

Conclusions

C2 nerve root coagulation and ligation with the self-irrigating bipolar sealer was safe and effective for maximizing exposure and minimizing EBL during atlantoaxial instrumentation among the selected patients. This novel technique provided optimal rapid exposure of the atlantoaxial joint, allowing accurate placement of instrumentation and direct decortication of the facet joints while demonstrating no vertebral artery injury or other neurovascular sequelae.

## Introduction

Posterior atlantoaxial fixation, first described by Goel and Laheri in 1994 and later modified by Harms and Melcher in 2001, is a highly successful operation for C1-2 stabilization [1-3]. However, safe and adequate exposure of the C1 lateral mass and C1-2 facet joint space can be obstructed by the bilateral C2 nerve roots and an accompanying perineural venous plexus. Typically, the C2 ganglion and venous plexus occupy more than 75% of the C2 foramen, obstructing access to the underlying bony anatomy [4]. Ligating the C2 nerve roots allows an unobstructed view of the C1 lateral mass and C1-2 joint space for safe screw entry, joint arthrodesis, and better fusion rates [5-9]. The theoretical benefits of C2 nerve ligation include shorter operative length and reduced blood loss when the venous plexus is coagulated and ligated along with the nerve roots [6,10]. On the other hand, retracting and preserving the C2 nerve roots prevents postoperative C2 hypesthesia and dysesthesia, which can occur in more than 10% of patients [11]. Additionally, neuropathic ulcers are a rare but significant complication of severe C2 hypesthesia [5]. However, preserving the C2 nerve roots has also been associated with postoperative C2 neuralgia in up to 33% of patients, which likely stems from a combination of nerve root manipulation, direct irritation by the C1 screw, and compression across the C1-2 joint space during arthrodesis [8]. Some surgeons have proposed that the use of intraoperative navigation may allow surgeons to safely instrument C1 with reduced blood loss while avoiding C2 nerve ligation; however, use of navigation is associated with increased operative time and cost [12]. Overall, studies have shown that C2 nerve ligation appears to provide safe and fast exposure of the C1-2 joint space for atlantoaxial fixation, although no consensus exists on nerve ligation versus preservation [5-10].

Techniques for C2 nerve ligation vary by surgeon. Conventionally, the C2 nerve root is coagulated using bipolar forceps and then tied with sutures or clipped to prevent cerebrospinal fluid (CSF) leaks and further bleeding. However, given the usual robustness of the venous plexus, conventional techniques can be time-consuming and may necessitate repeated coagulation throughout the procedure, causing delays in the C1-2 joint exposure. In this study, we investigated the hemostatic efficacy and safety of a commercial, self-irrigating bipolar sealer during ligation of the C2 nerve roots to shorten C1-2 facet exposure time and reduce blood loss during atlantoaxial fixation.

The abstract was presented at the Korean American Spine Society (KASS) 2024 in July 2024 at Park City, Utah.

## Materials and methods

Study design and patient population

This study was a retrospective analysis of a single-institution case series. All patients who underwent atlantoaxial fixation, either in isolation or as part of a longer construct, from May 2020 through July 2025 were eligible and were enrolled to minimize selection bias. Patients with prior hardware at C1 or C2 were excluded from the study. Patients consented to the surgical procedure and C2 nerve ligation with the self-irrigating bipolar sealer, as well as to data collection for research purposes. The HonorHealth institutional review board approved the retrospective investigation of the off-label use of the bipolar sealer. The study was conducted using the Strengthening the Reporting of Observational studies in Epidemiology (STROBE) guidelines [13].

Operative technique

**Figure 1:**
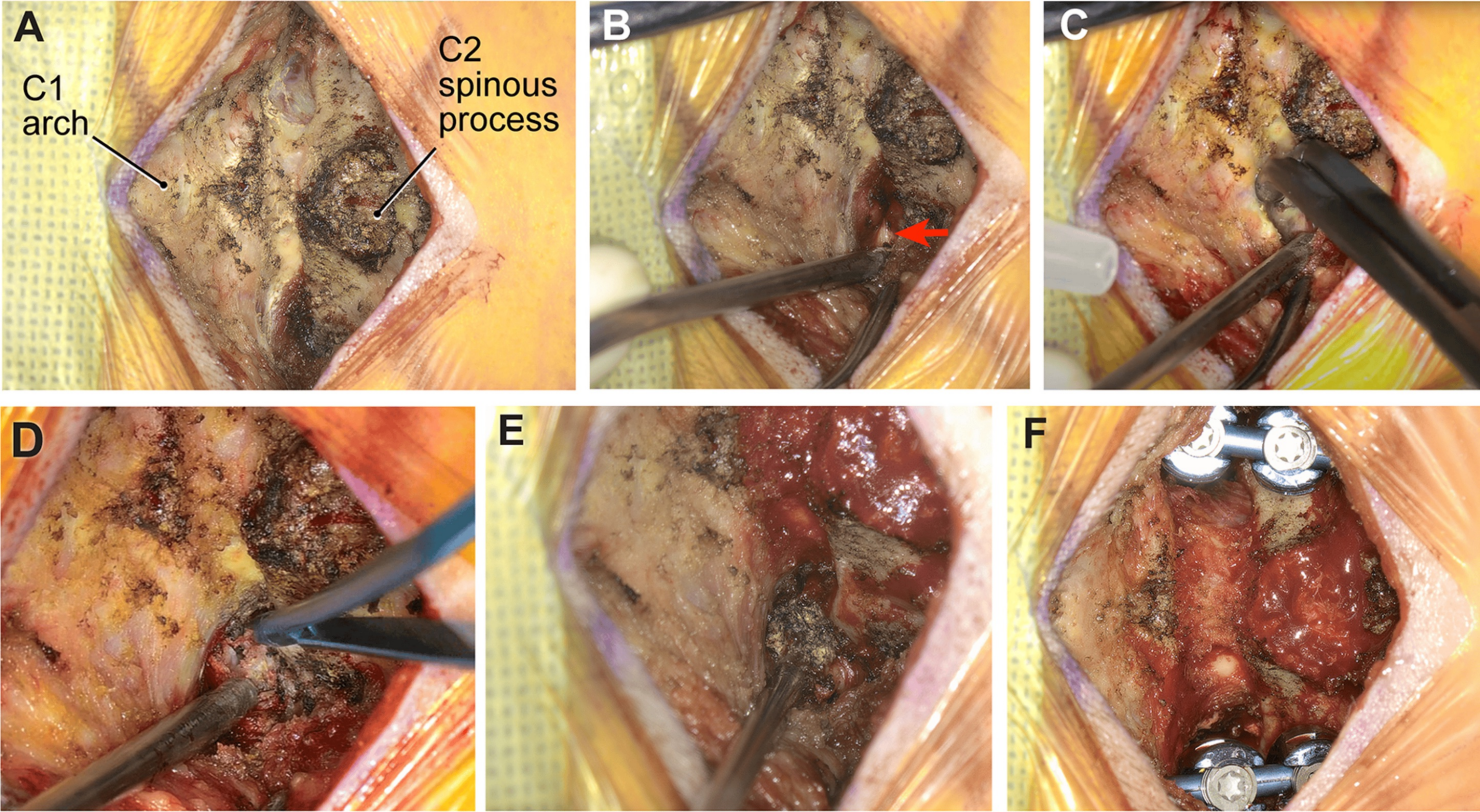
Intraoperative photographs of the atlantoaxial fixation procedure. (A) Standard midline exposure of the C1 posterior arch and C2 spinous process and laminae, showing the bilateral C2 foramen. (B) Retraction of the soft tissue, partially revealing the left C2 nerve root (red arrow). (C) Application of the bipolar sealer to the left C2 nerve root, proximal to the dorsal root ganglion (DRG). Cold saline irrigation is applied concurrently to minimize thermal damage. (D) Sharp resection of the C2 nerve root proximal to the DRG using microscissors. (E) After ligation of the C2 nerve root, the C1 lateral mass and the C1-2 facet joint space are exposed. (F) C1 lateral mass screws are placed under direct visualization. After C2 screws are placed, rods are used to connect the C1-2 screws. *Used with permission from Barrow Neurological Institute, Phoenix, Arizona.*

Patient positioning, surgical preparation, and draping are performed in the standard fashion, with the patient prone and their head stabilized in a three-point fixation device. After intraoperative localization of the C1 and C2 vertebrae using plain-film radiography, a linear midline incision is performed. After exposure of the atlantoaxial complex (Figure 1A), the soft tissue bundle containing the C2 nerve root and perineural venous plexus is identified but not fully dissected (Figure 1B).

**Figure 2:**
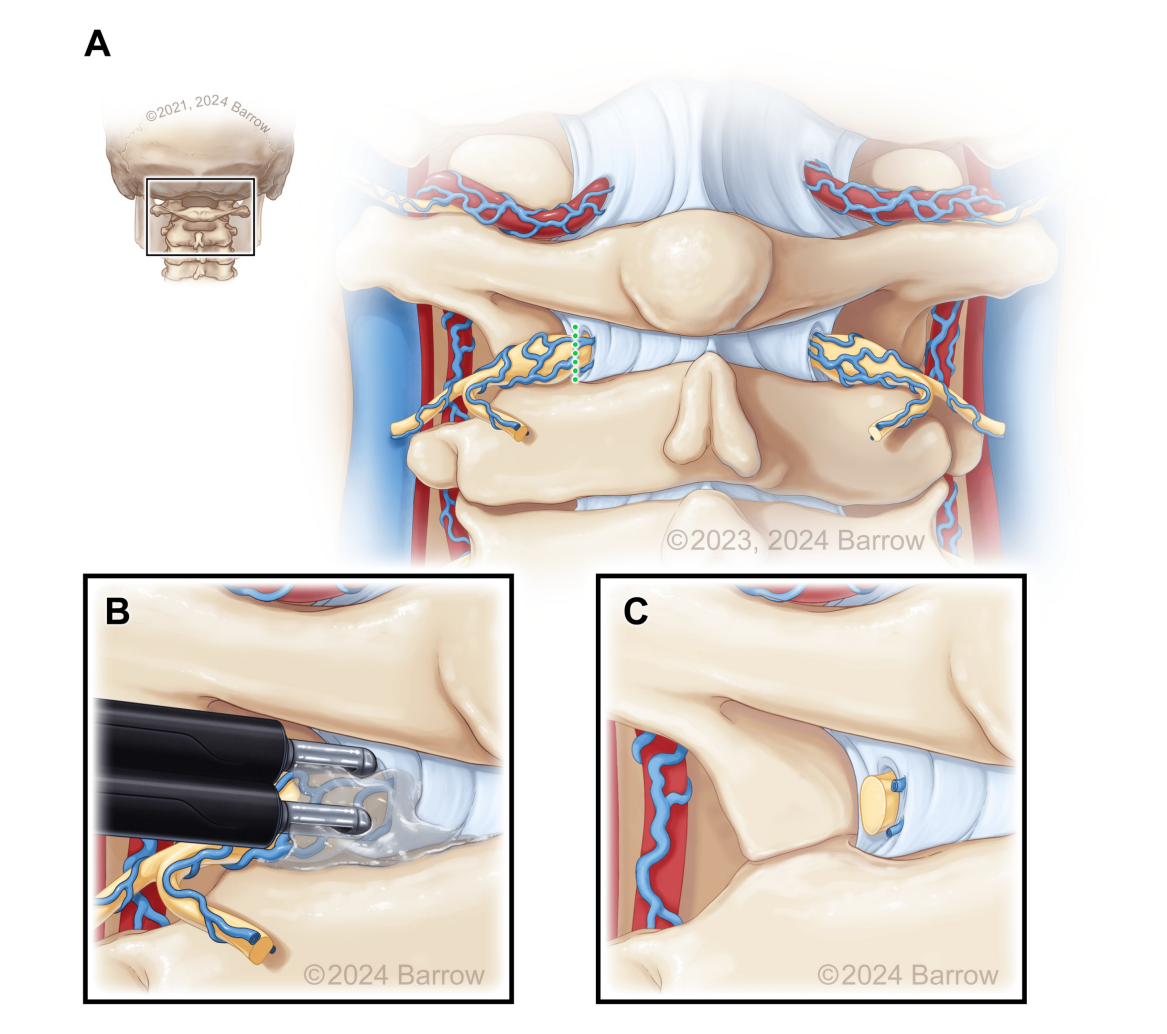
Illustration of C2 nerve root ligation using the self-irrigating bipolar sealer. (A) Occiput, C1, and C2 bony anatomy as shown in inset (black box), as well as the vertebral artery (red), C1 and C2 nerve roots (yellow), perineural and perivertebral venous plexus (blue), and location of ligation proximal to the dorsal root ganglion (DRG) (green dotted line). As shown, the C1 lateral mass is obscured by the C2 nerve roots bilaterally. (B) Placement of the bipolar sealer on the C2 nerve root proximal to the DRG, which is depicted by the bulbous enlargement of the C2 nerve roots. (C) Ligation of the C2 nerve root proximal to the DRG, which completely exposes the C1 lateral mass and the C1-2 facet joint space. *Used with permission from Barrow Neurological Institute, Phoenix, Arizona.*

The Aquamantys self-irrigating bipolar sealer (Medtronic, Dublin, Ireland) is set at 100-140 watts, and the nerve-venous complex is cauterized for 10-30 seconds per side as close to the takeoff from the thecal sac as possible to ensure ligation proximal to the C2 dorsal root ganglion (DRG) and away from the lateral vertebral artery (Figure 1C). The surgical assistant simultaneously applies cold saline irrigation to avoid thermal damage to the surrounding tissue. Then, microscissors are used to sharply resect the nerve root near the takeoff proximal to the DRG (Figure 1D). No clips or sutures are used to tie off the remaining stump. This technique allows the complete visualization of the underlying C1 lateral mass and the bilateral C1-2 joint space (Figure 1E). The exposed C1-2 joint spaces are decorticated, and arthrodesis is accomplished in the joint space and across the C1-2 posterior elements. C1 lateral mass screws and C2 pars and pedicle screws are then placed for fixation and fusion under direct visualization (Figure 1F) [14]. Figure 2 is an illustrative summary of the process.

For multilevel procedures, the other levels can be fixated using standard techniques. After fixation and arthrodesis, a drain can be placed, multilayer closure is performed, and cervical collars are placed on patients before leaving the operating rooms.

Data collection

The demographic and clinical data of the patients were collected retrospectively for the analysis. Clinic notes, hospital admission notes, progress notes, and discharge notes were reviewed to gather presenting symptoms, relevant imaging findings, postoperative examination results, and perioperative complications. Operative notes were reviewed to gather surgical data, including total surgical time, C1-2 exposure time, estimated blood loss (EBL), and intraoperative complications. Intraoperatively, the time from starting to use the self-irrigating bipolar sealer on the C1-2 foramen to the exposure of the bilateral C1-2 joint spaces after ligation of the C2 nerve-venous complex was separately tracked and recorded. The most recent clinic notes were reviewed for readmissions, reoperations, or other long-term complications.

Statistical analysis

All data are reported as mean (standard deviation (SD)) unless otherwise specified. No statistical analysis was necessary because there were no comparative groups.

## Results

Patient population

Twenty-seven patients (15 men, 12 women) with a mean age of 71.6 (8.6) years and mean body mass index 28.6 (6.4) underwent atlantoaxial fixation and ligation of the C2 nerve roots (Table 1).

**Table 1:** Demographic and clinical data for 27 patients who underwent atlantoaxial fixation and ligation of the C2 nerve roots with use of a self-irrigating bipolar sealer. Abbreviations: ASD, adjacent segment disease; BMI, body mass index; CSM, cervical spondylotic myelopathy; DF, dens fracture; EBL, estimated blood loss.

Subject ID	Age (years)	Sex	BMI	Pathology	Fusion length	Operative time (min)	C1-2 exposure time (min)	EBL (mL)	Length of stay (days)	Follow-up duration (months)
1	84.7	M	21.5	Trauma	C1-T3	228.0	3.7	300	12	16.7
2	66.9	F	30.1	Trauma	C1-3	96.0	3.9	200	3	16.7
3	79.3	M	26.0	CSM	C1-T1	244.0	3.6	200	16	14.8
4	66.4	M	32.2	Chronic DF	C1-2	97.0	4.7	100	4	11.5
5	73.6	M	36.8	Chronic DF	C1-3	98.0	2.8	50	2	8.0
6	73.6	F	25.3	ASD	C1-3	101.0	1.9	50	2	14.2
7	52.3	F	44.3	Trauma	C1-2, T2-5	173.0	2.5	200	23	6.1
8	73.9	F	30.2	Neoplasm	C1-3	174.0	2.1	200	6	6.5
9	51.8	F	24.9	Instability	C1-2	87.0	1.7	30	2	13.3
10	56.2	F	25.4	Neoplasm	C1-2	293.0	2.7	100	12	12.0
11	84.2	M	24.6	Chronic DF	C1-3	121.0	2.9	50	2	6.2
12	77.7	F	19.7	ASD	C1-4	77.0	2.4	100	2	14.6
13	81.3	M	23.0	Trauma	C1-2	62.0	2.5	75	8	12.3
14	76.0	M	30.4	Instability	C1-2	114.0	0.9	30	2	18.4
15	66.0	F	23.2	Instability	C1-2	125.0	1.5	50	2	6.8
16	68.6	M	32.3	ASD	C1-3	114	1.9	30	1	6.5
17	69.0	M	25.7	Pannus	C1-2	98	2.1	30	2	12.3
18	77.7	M	25.3	Instability	C1-2	130	1.8	50	3	13.1
19	71.9	M	31.1	Chronic DF	C1-2	88	2.0	50	1	13.5
20	72.9	M	24.1	Instability	C1-2	72	1.8	30	1	12.5
21	79.1	M	31.5	Instability	C1-2	72	1.8	50	1	6.5
22	74.1	F	26.2	Instability	C1-2	90	1.7	30	5	6.5
23	73.2	M	43.6	Pannus	C1-2	127	1.7	30	2	5.3
24	62.1	M	37.0	ASD	C1-4	122	2.0	50	4	1.5
25	75.8	F	33.7	Instability	C1-2	90	1.6	30	2	2.8
26	76.1	F	21.4	Trauma	C1-2	157	2.3	50	4	12.8
27	67.3	F	23.9	Trauma	C1-5	162	1.5	50	2	12.5
Overall, mean (SD)	71.6 (8.6)		28.6 (6.4)			126.4 (55.9)	2.3 (0.9)	82 (72)	4.7 (5.3)	10.5 (4.5)

The mean follow-up period was 10.3 (4.8) months. Fifteen patients (56%) underwent C1-2 fusion only, six patients (22%) underwent C1-3 fusion, and six patients (22%) underwent longer-segment fusion constructs. Six patients (22%) presented after an acute traumatic fracture of C1 or C2 or both; four patients (15%) presented with chronic C2 odontoid fractures, and the remaining 17 patients (63%) presented with other etiologies of cervical stenosis or instability. None of the patients had C2-distribution paresthesia, dysesthesia, or allodynia at presentation.

Operative data

The mean operative time for all 27 procedures was 126.4 (55.9) minutes, and the EBL was 82 (72) mL. For the 15 patients (55.5%) who had isolated C1-2 fusion, the operative time was 113.5 (55.9) minutes, and the EBL was 49 (25) mL. One of these patients presented with an intradural tumor at C1-2 that was resected. The mean operative time and EBL for the 14 patients (52%) with nonneoplasm C1-2 fusion were 100.6 (26.6) minutes and 45 (21) mL, respectively. The mean time from C2 nerve ligation to complete exposure of the C1-2 joint in all patients was 2.3 (0.9) minutes. There were no intraoperative complications or CSF leaks.

Postoperative outcomes

All patients were admitted to the hospital after surgery and had a mean length of stay of 4.7 (5.3) days. The 14 patients who had routine C1-2 fusion had a mean length of stay of 2.8 (1.9) days. At six weeks postoperatively, all patients experienced occipital or suboccipital numbness attributable to the C2 dermatome, but none of the patients reported C2 neuralgia or dysesthesia. All patients demonstrated radiographic stability, and their cervical collars were removed six to eight weeks postoperatively. At the most recent follow-up, all patients were neurologically stable with resolution of their preoperative symptoms, including upper cervical axial neck pain with rotation, without dysesthesia. One patient had a ground-level fall two years after the index surgery and required a revision for a broken C1 screw; no other patients needed revision surgery during the follow-up period.

Case example

**Figure 3:**
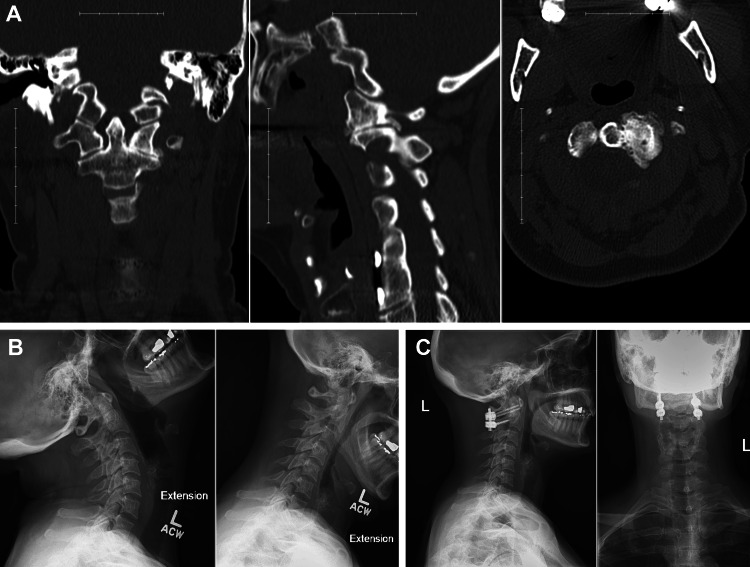
Case example of a patient in their early 50s with C1-2 spondylosis. (A) Plain computed tomographic images (left, coronal; middle, sagittal; and right, axial) of the cervical spine show greater degenerative spondylosis on the left than on the right of the C1-2 joint space. (B) Sagittal flexion-extension radiographs do not show overt instability in the cervical spine. (C) Postoperative six-week radiographs (left, sagittal; right, coronal) showing intact hardware and good alignment. *Used with permission from Barrow Neurological Institute, Phoenix, Arizona.*

A patient in their early 50s without a significant medical history or neurological deficits presented with progressive worsening of left-sided upper cervical neck pain (visual analog scale 7) radiating to the head on turning their head and was found to have significant left C1-2 facet joint spondylosis without significant dynamic instability (Figures 3A, 3B).

Upon receiving a left C1-2 facet joint injection, the patient had complete relief of the pain symptoms for two weeks. Due to the failure of the injection to provide lasting relief, the decision was made to proceed with atlantoaxial fixation. Intraoperatively, we used the technique described above to expose the C1-2 joint space bilaterally for rapid and safe placement of screws. Arthrodesis was achieved. The patient tolerated the procedure well, and the six-week postoperative radiographs demonstrated intact hardware and optimal alignment (Figure 3C). The patient reported expected numbness in the C2 dermatome without dysesthesia or deficits and resolution of their preoperative cervical pain symptoms (visual analog scale 1).

## Discussion

In this retrospective case series, we investigated the novel use of a commercially available, self-irrigating bipolar sealer to prepare the C2 nerve roots for ligation during atlantoaxial fixation. We found that, compared with preserving the C2 nerve roots or using conventional bipolar forceps to cauterize the nerve roots, the use of this bipolar sealer resulted in a quick exposure of the C1-2 space in 2.3 (0.9) minutes, with minimal EBL of 82 (72) mL and without perioperative or long-term complications. Historically, prior to the use of the self-irrigating bipolar sealer, when we used conventional bipolar forceps in over 50 patients, our EBL for a C1-2 was approximately 200 (50) mL, and the C1-2 joint exposure took approximately 45 (15) minutes.

Direct visualization of the C1 lateral mass and the C1-2 facet joint facilitates safe instrumentation during atlantoaxial fixation. Inadequate exposure of the anatomy could potentially lead to misplacement of the C1 and C2 screws and increased risk of severe neurovascular injuries. Although more recent advances in 3D neuronavigation techniques can help avoid misplacement, they add time and cost to the surgery [12]. The C2 nerve root occupies approximately 75% of the C2 foramen and makes full exposure of the underlying C1 lateral mass and C1-2 facet joints difficult [4]. In contrast to C2 nerve root ligation, retracting the C2 nerve roots and placing C1 screws can often lead to C2 neuropathy. An additional advantage of C2 nerve ligation lies in the subsequent ability to completely arthrodese the C1-2 joint. Exposure of the C1-2 joint space is greatly increased after C2 nerve ligation, allowing complete decortication of the bilateral joints and improved arthrodesis [4,8,9]. Without C2 nerve ligation, the surgeon relies on posterior element arthrodesis without facet arthrodesis, which may reduce fusion rates [15]. Thus, C2 nerve ligation has the potential to increase the efficacy of C1-2 arthrodesis while minimizing blood loss and operative time. Many studies have shown the potential benefits of cauterizing and ligating the C2 nerve roots, but there is no consensus on the optimal method for performing this ligation [5-8].

The self-irrigating bipolar sealer is often used in spine surgery to aid in hemostasis [16-18]. Unlike traditional bipolar forceps, the self-irrigating bipolar sealer lacks fine control at the tips as the two bipolar prongs are fixed in space. However, the self-irrigating bipolar sealer offers improved hemostatic capabilities as its electric output can be increased significantly while minimizing the surrounding tissue damage with irrigation. When ligating the C2 nerve roots with traditional bipolar forceps, the surgeon often needs to dissect and isolate the nerve-venous complex from the surrounding soft tissue and bone because the narrow prongs and low power of the bipolar forceps may not fully cauterize the vein and nerve. Furthermore, the surgeon must often clip or tie off the nerve root sleeve before ligation to avoid CSF leakage. In contrast, the surgeon can cauterize the C2 foraminal space more generally with the self-irrigating bipolar sealer. Its wide, blunt, fixed prongs are unlikely to cause inadvertent injury, and its high power completely cauterizes the nerve-venous complex. Also, because the self-irrigating bipolar sealer effectively seals the target tissue, there is no need for separate clipping or tying of the nerve root sleeve, and we observed no evidence of CSF leaks intraoperatively or postoperatively. Furthermore, in our experience, the continuous irrigation during hemostasis prevents the coagulated tissue from adhering to surgical instruments during subsequent steps, which appears to reduce the incidence of residual hemorrhage during tissue manipulation. Overall, we found the bipolar sealer to be safe, effective, and efficient for ligation of the C2 nerve roots in our patients, allowing quick exposure of the bony anatomy required for atlantoaxial fixation and arthrodesis, with minimal blood loss.

Our results demonstrated the safety and efficacy of ligating the C2 nerve roots during atlantoaxial fixation and fusion among our selected patients. Our results are consistent with prior literature showing that C2 nerve sacrifice does not lead to insurmountable adverse outcomes. Further studies are necessary to evaluate whether the reduction in operative time and EBL associated with using the bipolar sealer for atlantoaxial fixation offset the additional cost of the equipment. Additionally, further studies of clinical and radiographic outcomes to assess quality-of-life measures and fusion rates are needed to delineate the additional benefits of the self-irrigating bipolar sealer and novel technique we describe.

Limitations

This study reports a retrospective, noncontrolled case series with a small sample size and relatively short follow-up and was performed at a single institution. Thus, the generalizability of its findings is limited, and comparisons with the current bipolar cauterization technique cannot be performed. Furthermore, a detailed sensory analysis of the C2 dermatome was not performed during this preliminary investigation. Thus, the effects of our technique on postoperative C2 dysesthesia and numbness cannot be compared with existing literature. Nonetheless, our technique was used in all patients undergoing atlantoaxial fixation, reducing patient selection bias. This study has established the potential safety of this technique and lays the groundwork for future studies involving direct comparison between our technique and the traditional bipolar technique.

## Conclusions

The use of the Aquamantys self-irrigating bipolar sealer to prepare for the ligation of the C2 nerve roots during atlantoaxial fixation has not been previously reported. In this series of 27 patients who underwent atlantoaxial fixation, we demonstrated the feasibility, safety, and efficacy of this novel application of the bipolar sealer to achieve rapid exposure of the C1-2 bony anatomy with minimal blood loss in our series of patients. Larger studies with longer-term follow-up are necessary to determine whether the clinical outcomes of this novel technique are comparable to those of existing methods.
